# Pneumocystis jirovecii pneumonia and deep vein thrombosis in a patient with glioblastoma multiforme

**DOI:** 10.15190/d.2022.20

**Published:** 2022-12-31

**Authors:** Hussain Hussain, Michael Paidas, Aya Fadel, Efrain Garcia, Zahraa Saadoon, Luis Mendez, Arumugam Jayakumar

**Affiliations:** ^1^Larkin Community Hospital, Department of Internal Medicine, Miami, FL, USA; ^2^University of Miami Miller School of Medicine Department of Obstetrics, Gynecology and Reproductive Sciences, University of Miami Miller School of Medicine, Miami, FL, USA; ^3^Department of Internal Medicine at Ocean Medical Center, Brick City, New Jersey, USA; ^4^University of Baghdad School of Medicine, Baghdad, Iraq

**Keywords:** Vancomycin, bactrim, thrombosis, shock, pneumocystis jirovecii pneumonia, glioblastoma multiforme.

## Abstract

We present a case of disseminated Pneumocystis jirovecii pneumonia in a patient with a medical history of glioblastoma multiforme associated with acute deep-vein thrombosis. The patient presented to the emergency department with clinical features of pulmonary infection, and the chest images showed pneumonia. Antibiotics were initiated (azithromycin, cefepime, and vancomycin) and the patient was transferred to the ward for further management, where the condition of the patient continued to worsen over the second day. The patient developed bilateral lower extremity swelling and the doppler ultrasound revealed bilateral lower extremity acute deep vein thrombosis. Laboratory results showed pancytopenia and transaminitis. However, a repeated chest X-ray showed ground-glass changes and interstitial infiltrates, suggestive of atypical infection. We indeed identified D-glucan which hints to a disseminated form of Pneumocystis jirovecii pneumonia infection in this patient. We further confirmed the Pneumocystis jirovecii pneumonia by polymerase chain reaction test from the fluid obtained via bronchoalveolar lavage. We, therefore, initiated intravenous trimethoprim/ sulfamethoxazole treatment with an anticoagulant, and the patient’s condition improved. Our findings strongly suggest a possible link between Pneumocystis jirovecii pneumonia infection and thrombogenesis, with impact in medical practice.

## INTRODUCTION 

Pneumocystis jirovecii Pneumonia (PCP) is a life-threatening fungal infection in patients with immunocompromised conditions, such as acquired immunodeficiency syndrome (AIDS), leukemic and organ transplant recipients. Immunocompetent patients with PCP are usually asymptomatic^1-3^. In most cases, PCP resides in the lung, and it invades multiple organs, including bones, liver, and the central nervous system^2,3^. There are specific factors that can potentially lead to the development of thrombogenesis; 1) immobility, 2) increase venous pressure, 3) mechanical injury to the vein, 4) increase blood viscosity, 5) anatomical abnormality, 6) genetic factors leading to recurrent thrombosis (e.g, protein C, S, anti-thrombin III deficiencies, and factor V Leiden mutation)^4^. Pathophysiology is classified according to Virchow’s triad: damage to the vessel wall, blood flow turbulence, and hypercoagulability^4^. Multiple factors trigger such triad including cytokines, chemokines, endothelial damage, and idiopathic^4^. In acute infection there is a specific surge for cytokines and chemokines can trigger the development of thrombosis^5^. A previous study suggested a possible link between PCP infection and thrombosis. However, other factors/complications identified in this patient may have been involved in the development of deep vein thrombosis (DVT). In addition, another case of PCP infection associated with the development of pulmonary embolism (PE) was identified by Titilope et al.^6^. Our report now brings additional proof about the possible link between PCP infection and DVT development, since in our case we do not see additional complications, other than disseminated acute PCP infection on a background of glioblastoma multiforme. 

## CASE PRESENTATION 

**Figure 1:**
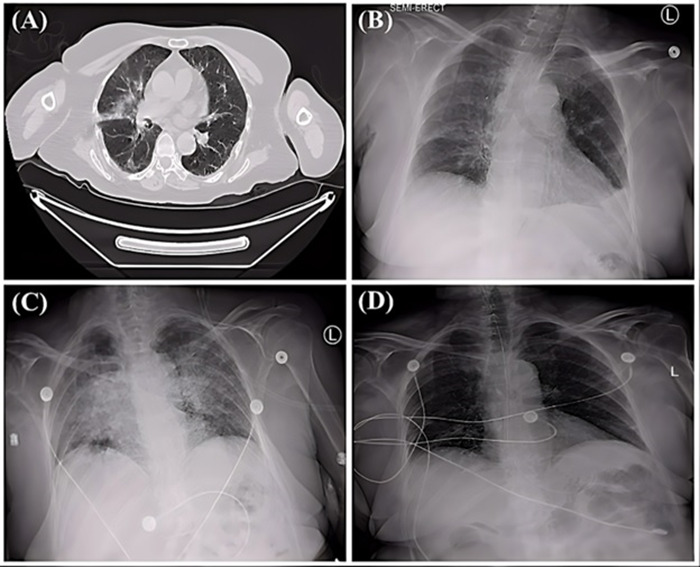
Radiographical findings **A.** Chest CT revealed bilateral multifocal ground glass opacities notably of the right upper lobe, reactive nodules, and bibasilar reticular densities left more than right.** B.** Chest X-ray showed infiltration and ground glass opacities. **C.** Chest X-ray revealed severe bilateral infiltration.** D.** Chest X-ray showed improvement after bactrim treatment.

A 71-year-old female with a past medical history of glioblastoma multiforme (GBM) was treated with a gamma knife and temozolomide from January - July 2021 and developed disseminated PCP infection. In early August 2022, the patient was completely asymptomatic, and her laboratory results and physical examination were within normal limits. In mid-August 2022, the patient came to the emergency department (ER) complaining of three days of low-grade fever, night sweating, and fatigue along with a productive cough with green sputum. Later, the patient was diagnosed in the ER with sepsis according to systemic inflammatory response syndrome (SIRS) criteria, which was stabilized with intravenous fluid and antibiotics azithromycin, cefepime, and vancomycin. Blood culture was negative, and chest computer tomography (CT) scan disclosed pneumonia (Figure 1), while the result of complete blood count (CBC) and comprehensive metabolic panel (CMP) are shown in Table 1. 

**Table 1:** Baseline Patients' Characteristic White blood cells (WBC); red blood cells (RBC); aspartate aminotransferase (AST); alanine transaminase (ALT); lactate dehydrogenase (LDH).

Type of parameter along with the normal range	Day 1	Day 2	Day 3	Day 5	Day 6	Day 7	Day 9	Day 10
WBC (Normal range is 5000-11000 microliter)	5530	5.0	4.1	3.8	2.9	2.8	2.8	2.3
RBC (Normal range is 4-6 microliter)	3.1	2.7	2.4	2.1	1.8	1.9	1.82	1.79
Hemoglobin (Normal range is 12-17 g/dl)	10.1	9.4	9.2	9	8.79	8.8	8.4	8.1
Platelets (Normal range is 150,000-450,000 microliter)	119,700	117	120	115	66	54	37	21
AST (Normal range is 8-33 U/L)	78	85	98	119	187	201	205	213
ALT (Normal range 4-36 U/L)	71	88	91	113	129	186	193	202
LDH (Normal range is 105-333 IU/L)	411	488	470	498	465	501	480	487
Reticulocytes (Normal range is 0.5-2.5 %)	0.43	0.41	0.38	0.42	0.32	0.37	0.39	0.4
PaO2 (Normal 80-100 mmHg)	71	69	66	70	61	58	77	75
D-dimer (Normal range is <0.5 g/l)	2.1	2.1	3.9	2.6	4.5	5.1	5.5	7.9

**Figure 2:**
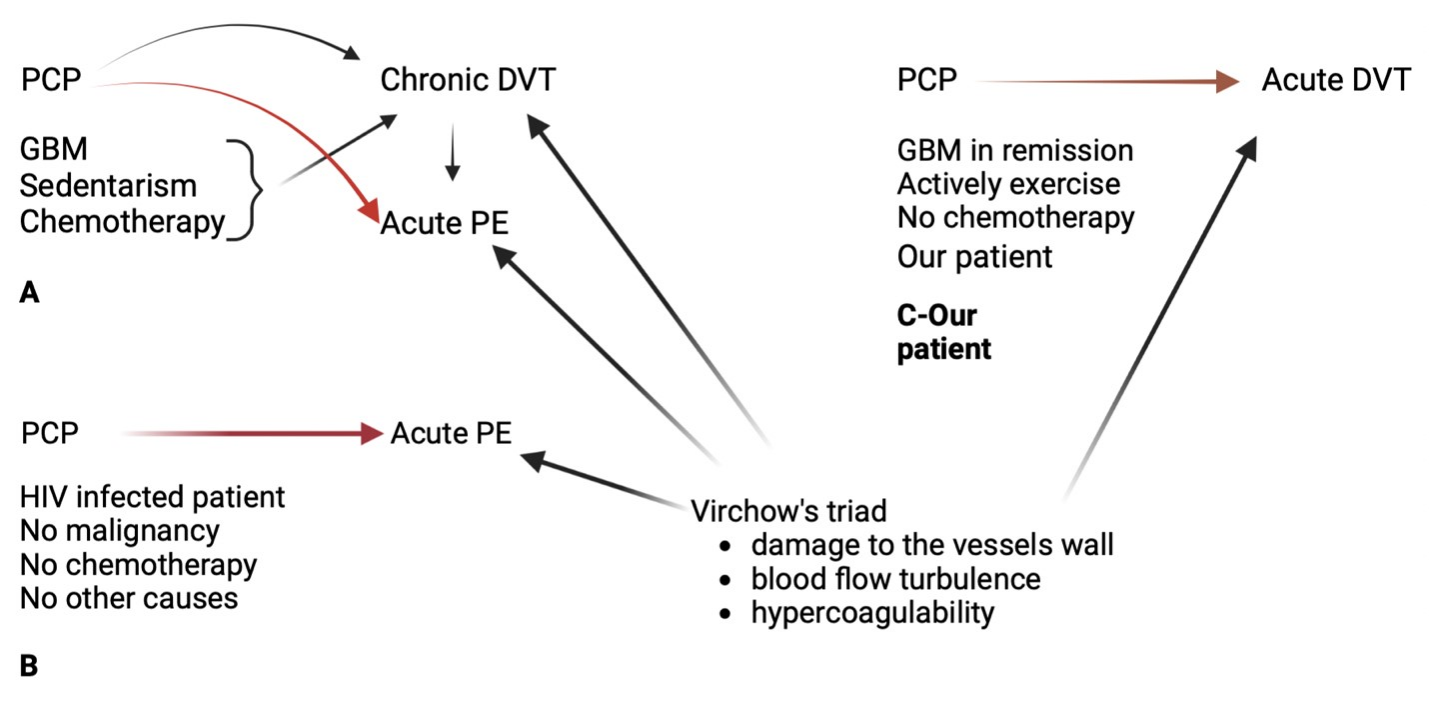
Three different patients presenting with PCP infection and thrombi **A**. Multiple factors in addition to the PCP contributed to the development of chronic DVT and acute PE. **B**. HIV-positive patient who presented acute PCP and PE simultaneously with no other complications. **C**. While our patient presented PCP and acute DVT, the GBM was in remission and had no chemotherapy or sedentarism. Virchow’s triad explains the pathophysiology of thrombogenesis. Pneumocystis jirovecii pneumonia (PCP), glioblastoma multiforme (GBM), deep vein thrombosis (DVT), pulmonary embolism (PE), Human Immunodeficiency Virus (HIV).

The erythrocyte sedimentation rate (ESR) was elevated at 96 mm/h. The sputum culture and urine culture were negative. The aspartate aminotransferase (AST), and alanine transaminase (ALT) were elevated, as well as lactate dehydrogenase (LDH) (Table 1). The patient's condition was not improved on the antibiotics. The cough became non-productive with mild shortness of breath, fatigue, nighttime chills, and bilateral scattered ecchymosis over the extremities noted. Repeated chest x-ray showed diffused bilateral infiltration, and laboratory results display trending down of both RBC, WBC, and platelets (Table 1). Reticulocytes were mildly low (Table 1). AST and ALT continued to trend up (Table 1). The coagulation study disclosed elevated D-dimer (Table 1), while the prothrombin time (PT) along with partial thromboplastin time (PTT) were within normal limits. Based on the clinical and radiographical deteriorations (ground-glass changes and interstitial infiltrate) along with the laboratory abnormalities, we suspected a disseminated PCP. The D-glucan was detected. Thus, bactrim 20 mg/kg was initiated intravenously twice a day. The PCP was confirmed by polymerase chain reaction (PCR) on the fluid obtained from the lung by bronchoalveolar lavage (BAL). The patient complained of bilateral acute lower extremity swelling and pain, doppler ultrasound showed acute bilateral deep vein thrombosis (DVT). Thus, enoxaparin 1 mg/kg was added to the regimen, which resulted in laboratory-confirmed heparin-induced thrombocytopenia (HIT) as the platelets dropped to 66,000 (day 6) and continued to decrease, hence, enoxaparin was suspended. The rest of the infectious investigation (viral hepatitis, aspergillosis, mycobacterium, histoplasmosis, PCP, cytomegalovirus (CMV), Epstein-Barr virus (EBV), legionella, streptococcus, mycoplasma, and human immunodeficiency virus (HIV)) were negative. The clinical, laboratory and radiographical findings were significantly improved (Figure 1) when the patient was on bactrim. Also, dexamethasone was added according to the protocol, and a low dose of fondaparinux treatment was initiated since the platelet level increased to 81,000, then the dose of fondaparinux was increased as per the guideline. A few days later, the patient returned to the baseline condition. The DVT continued to improve by clinical and radiographical findings, and after 7-8 weeks the doppler ultrasound confirmed the resolution of DVT. Patient developed a chronic DVT and presented to the ER with the acute setting of PCP infection concomitantly associated with acute PE, the patient had multiple events such as active GBM, sedentarism, and chemotherapy were contributed to the development of thrombogenesis, yet PCP may be playing an important role as well^7,8^. Patient C had a history of HIV and presented to the ER with acute PCP infection and acute PE, no risk factors were identified in that patient besides the active infection of PCP^6^. Further, our patient developed acute PCP infection simultaneously with acute DVT. All patients share the characteristic of Virchow’s triad in the development of thrombosis (Figure 2). The authors mentioned in their published studies the simultaneous association of PCP infection with thrombosis, yet no pathophysiology was included in either paper^6,8^. We think there is a surge in blood cytokines/chemokines that may contribute to the development of thrombosis. All patients were treated appropriately with medications (antibiotics and anticoagulants) led to the resolution of conditions.

## DISCUSSION

The development of acute thrombi was diagnosed only in two prior cases that concomitantly infected with acute PCP. Olanipekun, T. et al, reported a case of acute PCP infection in HIV positive patient concomitantly presented to the ER with acute PE^6^. The author mentioned the simultaneous association between the development of acute PE and PCP infection, no other factors were presented in that patient to aggravate the thrombogenesis^6^. Braiteh F, and Nash I, narrated a case of acute PCP infection in a patient who developed acute pulmonary embolism (PE), along with chronic deep vein thrombosis (DVT)^7^. Lastly, we diagnosed a patient with acute PCP infection who developed acute bilateral lower extremity DVT without multiple events that contributed to- or aggravated the thrombogenesis. Noteworthy, our patient’s malignancy was treated adequately, the patient was in remission and no chemotherapy was taken. Furthermore, clinical and laboratory investigation before the development of PCP in our patient was completely normal. All those patients including our patient share similarities in the pathogenesis of thrombi since Virchow’s triad is presented in all patients^8-13^ (Figure 2). 

The incidence of PCP has declined in the last 5-10 years due to advanced diagnosis and treatment of HIV when PCP was highly associated with such a condition^1,2,13^. The PCP is potentially an inhabitant of the lung, but it can be spread to different organs^1,2,13^. Initially, the PCP is attached to the type I pneumocyte, which induces a severe inflammatory response through the activation of various cytokines and chemokines^2,12,13^. Such inflammation can damage the alveoli. As far as the PCP disseminated, the severity of the inflammation worsened through producing various cytokines (interleukin 1 beta, tumor necrosis factor, gamma interferon, etc.). Which in turn may lead to severe complications including sepsis and coagulopathy^2-4,13^. 

Disseminated PCP in our patient and in patient B was likely triggering various inflammatory cytokines and dysfunction of endothelial cells, which have critical roles in the regulation of coagulation, release cytokines that stimulate T-cells, as well as regulating permeability and vascular tone^4-6,8,12^. Endothelial cell dysfunction plays a critical role in thrombogenesis. In a murine model of DVT, dysfunctional endothelial cells due to inflammation are shown to increase the expression of adhesion molecules that aid in the attachment of leukocytes and platelets to the endothelium^9,10^. Moreover, the leukocyte integrin Mac-1 may facilitate the adhesion process of the platelets to endothelium by Platelet glycoprotein Ib alpha (GPIb-alpha)^5,9,10^. And the leukocytes can adhere to the endothelium by intracellular adhesion molecule-1 (ICAM). In addition, the platelet C lectin-like receptor (CLEC-2) released from endothelium can potentially trigger thrombogenesis, which has been confirmed in mice studies with knocked-out CLEC-2 molecules, which reveals no thrombus formation in those mice^9,10^. The current study may suggest a link between PCP infection and thrombosis formation. PCP infection may trigger a systemic inflammatory response leading to the development of thrombi. Furthermore, it should be highlighted that interleukins, interferon-gamma inducible protein (CXCL-8), monocyte chemoattractant protein (CCL-2), tumor necrosis factor (TNF-alpha), and gamma interferon are considered as pro-thrombotic factors^9-11^ and the pro-inflammatory cytokines and adhesion molecules (selectins L, E, and P) were identified to be associated with venous thrombosis in various studies^4-7,9-11,14^. Additionally, the level of P-selectin was also increased in patients with thrombi during infection^9-11^. This may indicate the potential involvement of P-selectin in thrombogenesis by direct or indirect effect through stimulation of various inflammatory cells to accumulate and aggravated endothelial injury^8-10^, although additional investigations are warranted. 

## CONCLUSION

Disseminated PCP can lead to various complications such as sepsis, hepatitis, and neurological manifestations. Three patients were diagnosed with acute PCP infection and developed thrombi, these events interpret an important likelihood between thrombogenesis and acute PCP infection. Generally, there are various triggers for the development of thrombi, the most important in those cases is the acute inflammation that triggers the release of various cytokines and chemokines that contributed to thrombogenesis. Future investigation is warranted to elaborate the mechanism of possible thrombogenesis and PCP infection.
